# An empirically based conceptual framework for fostering meaningful patient engagement in research

**DOI:** 10.1111/hex.12635

**Published:** 2017-10-06

**Authors:** Clayon B. Hamilton, Alison M. Hoens, Catherine L. Backman, Annette M. McKinnon, Shanon McQuitty, Kelly English, Linda C. Li

**Affiliations:** ^1^ Department of Physical Therapy University of British Columbia Vancouver BC Cananda; ^2^ Arthritis Research Canada Richmond BC Canada; ^3^ BC SUPPORT Unit Vancouver BC Canada; ^4^ Arthritis Patient Advisory Board Arthritis Research Canada Richmond BC Canada; ^5^ Department of Occupational Science & Occupational Therapy University of British Columbia Vancouver BC Canada

**Keywords:** conceptual framework, patient and public involvement, patient engagement in research, patient‐oriented research

## Abstract

**Background:**

Patient engagement in research (PEIR) is promoted to improve the relevance and quality of health research, but has little conceptualization derived from empirical data.

**Objective:**

To address this issue, we sought to develop an empirically based conceptual framework for meaningful PEIR founded on a patient perspective.

**Methods:**

We conducted a qualitative secondary analysis of in‐depth interviews with 18 patient research partners from a research centre‐affiliated patient advisory board. Data analysis involved three phases: identifying the themes, developing a framework and confirming the framework. We coded and organized the data, and abstracted, illustrated, described and explored the emergent themes using thematic analysis. Directed content analysis was conducted to derive concepts from 18 publications related to PEIR to supplement, confirm or refute, and extend the emergent conceptual framework. The framework was reviewed by four patient research partners on our research team.

**Results:**

Participants’ experiences of working with researchers were generally positive. Eight themes emerged: procedural requirements, convenience, contributions, support, team interaction, research environment, feel valued and benefits. These themes were interconnected and formed a conceptual framework to explain the phenomenon of meaningful PEIR from a patient perspective. This framework, the PEIR Framework, was endorsed by the patient research partners on our team.

**Conclusions:**

The PEIR Framework provides guidance on aspects of PEIR to address for meaningful PEIR. It could be particularly useful when patient‐researcher partnerships are led by researchers with little experience of engaging patients in research.

## 1 INTRODUCTION

Patient engagement in research (PEIR) involves patients or their surrogates undertaking roles beyond those of traditional study participants along the continuum of the research process, including knowledge translation. This practice enables patients to contribute their perspectives to research.^1^, ^2^, ^3^, ^4^, ^5^ Integrating a patient perspective is meant to influence the creation, dissemination and use of evidence‐based knowledge to reflect and meet the health‐care needs and preferences of patients.^6^ PEIR could therefore potentially increase the quality and appropriateness of research and ultimately improve health‐care services.^7^ The term *patient and public involvement*, commonly used in the UK,^6^ is a synonym of PEIR.

The increased popularity of PEIR in the last 15 years coincides with a greater awareness among funding agencies that patients are key stakeholders of health research.^6^, ^8^, ^9^, ^10^ Its impacts have been categorized within three‐value systems that cover the “moral, ethical and political concerns,” “consequences” and “process” of PEIR.^7^ Emerging evidence supports the theory that engaging patients in research can improve the quality and relevance of research.^11^, ^12^ Unfortunately, there are no controlled evaluations of methods for PEIR.^12^, ^13^ This is possibly because there is limited conceptualization or theoretical development that would underlie evaluation tools, which has hindered its evaluation.^12^, ^14^ Recent systematic reviews on the engagement of patients as research partners have highlighted that the practice was generally tokenistic, poorly reported and under‐reported.^12^, ^15^

The literature provides several key attributes of PEIR as guiding principles and components of models.^1^, ^6^, ^9^, ^10^, ^16^ The guiding principles for fostering *meaningful* engagement of patients include involving patients early (contributing to research questions and design), inclusiveness, co‐learning, co‐building of knowledge and providing support.^6^, ^9^, ^10^ Two models, one in arthritis and the other in breast cancer, have been identified in the earlier literature in this field,^1^, ^16^ both of which were developed by teams of patients and researchers using their own experiences of engaging in research.^1^, ^16^ Neither of these models, however, was originally developed through empirical studies. Using an empirical approach can advance the conceptualization of PEIR by systematically collected data to build on anecdotal knowledge.

While we found no studies explicitly exploring this phenomenon, “meaningful PEIR” does appear frequently in the health‐care literature.^9^, ^10^, ^17^, ^18^ For example, besides attributes already mentioned, the National Health Council asserts that meaningful PEIR should benefit both *patient partners* and researchers and be an informative and constructive process.^18^ Patient partners are the patients and informal caregivers, such as their family members and friends, who engage to provide a patient perspective in health research. One empirical study developed a conceptual framework that outlines the broader phenomenon of successful interprofessional collaborations in health‐care research but does not have a complete focus on the role of patients.^19^ The value of a conceptual framework is that it links “concepts that together provide a comprehensive understanding of a phenomenon” (p. 57).^20^

This study aimed to develop a conceptual framework for meaningful PEIR from a patient perspective. The practical value of this framework would include the use of its components to guide the planning, implementing and evaluating of PEIR. We formed a researcher‐initiated collaboration between four experienced patient partners, all of whom are living with arthritis (AMH, AMM, KE and SM), and three health‐care researchers (CBH, CLB and LCL). AMH is also an experienced knowledge broker and a registered physiotherapist.

## 2 METHODS

We conducted a qualitative secondary analysis of interview data from a study involving patient partners using a thematic analysis approach. The patient partners on our team were female adults from the Arthritis Patient Advisory Board (APAB) of Arthritis Research Canada who have engaged in several completed and on‐going health research projects. CLB is an outcomes researcher with expertise in qualitative research. LCL is a health services researcher with expertise in knowledge translation and implementation science. The University of British Columbia Behavioural Research Ethics Board approved this study (REB#H15‐00217).

### 2.1 Data sources

Interview data were collected through *informal data sharing* from a study exploring patient partners’ views and experiences of engaging in research, and understanding the barriers to and facilitators of their engagement.^21^, ^22^ In that study, a purposive sample of 22 patients with arthritis, all past or current members of APAB, participated in one‐on‐one in‐depth interviews (averaging 1 hour) co‐facilitated by two interviewers during August‐November 2015.^21^ APAB members are patient volunteers who offer their perspectives and expertise to the health research process, including knowledge translation and governance. The participants completed a demographic form about their age, gender, education level, employment status, length of APAB membership, number of projects engaged in and health conditions. Eighteen participants, assigned self‐selected pseudonyms, consented to their de‐identified and personally verified transcripts being included in the present analysis. These interview data were appropriate for the aim of the present study because the interviews elicited patient experiences as patient partners in a variety of projects and the interview guide^21^ sought their suggestions for what would (and would not) help to advance their engagement in research. For example, the interviewers used probes such as “What do you like or dislike about being involved in research?,” “Thinking about your experience, could you tell me what helps you take part in research” and “To what extent do you feel your contributions are valued by researchers?”^21^

To integrate the emergent themes from the interviews with established concepts about PEIR, CBH selected 18 key publications related to PEIR from the scientific and grey literature (Table 1).^1^, ^4^, ^6^, ^9^, ^10^, ^11^, ^13^, ^15^, ^16^, ^19^, ^23^, ^24^, ^25^, ^26^, ^27^, ^28^, ^29^, ^30^ These publications presented guiding principles, frameworks, models and recommendations relevant to engaging patients, communities, service users, stakeholders or the public in research.^1^, ^4^, ^6^, ^9^, ^10^, ^11^, ^13^, ^15^, ^16^, ^19^, ^23^, ^24^, ^25^, ^26^, ^27^, ^28^, ^29^, ^30^ They were used in our analysis to supplement, confirm or refute the themes arising from the interviews.

**Table 1:** Key publications related to patient engagement in research

First author, date and title of publications	Highlights
Howe (2017)^29^ Learning to work together—lessons from a reflective analysis of a research project on public involvement	Provided recommendations as ingredients for good patient and public engagement in research
Cheung (2016)^25^ Recommendations for the involvement of patient research partners (PRP) in OMERACT working groups. A report from the OMERACT 2014 working group on PRP	Provided a list of eight recommendations for patient engagement in research projects by the working groups of Outcome Measures in Rheumatology (OMERACT)
Kirwan (2016)^13^ Emerging guidelines for patient engagement in research	Provided a list of six guidelines for engaging patients as patient research partners in outcomes research
Johnson (2016)^26^ The patient voice in research—evolution of a role	Provided a model for successfully engaging patients in research, developed by patient research partners who engaged in a breast cancer study
Forsythe (2016)^23^ Patient and stakeholder engagement in the PCORI pilot projects: description and lessons learned	Provided themes on lessons learned on engaging patient and stakeholders in pilot projects funded by Patient‐Centered Outcomes Research Institute (PCORI)
de Wit (2015)^16^ Do not forget the professional—the value of the FIRST model for guiding the structural involvement of patients in rheumatology research	Provided updates to the FIRST (facilitate, identify, respect, support, train) model to guide successful collaboration between patients and researchers
Esmail (2015)^28^ Evaluating patient and stakeholder engagement in research: moving from theory to practice	Provided an overview of the then current state of the measurement of hypothesized impact of patient engagement in research
Shippee (2015)^15^ Patient and service user engagement in research: a systematic review and synthesized framework	Provided a framework for reporting patient and user engagement in research
Frank (2015)^9^ Conceptual and practical foundations of patient engagement in research at the Patient‐Centered Outcomes Research Institute	Conceptual model of patient‐centred outcomes research
Soever (2014)^19^ Collaborative inter‐relational health‐care research: a conceptual framework informed by a qualitative enquiry	Provided a conceptual framework for successful interprofessional collaborations in health‐care research
Brett (2014)^34^ Mapping the impact of patient and public involvement on health and social care research: a systematic review	Provided themes for evidence of the beneficial impacts and challenging impacts of patient and public engagement in health and social care research
Canadian Institutes of Health Research (2014)^10^ Strategy for patient‐oriented research—patient engagement framework	Provided four guiding principles for patient engagement in research
EUALR (2013)^30^ Patient involvement in research—a way to success	Provided six reference cards for both researchers and PRPs to improve their collaboration
Hayes (2012)^6^ Briefing notes for researchers: public involvement in NHS, public health and social care research	Provided briefing notes to researchers on how to engage patients and the public in research, with specific guidance for each research stage
Staniszewska (2011)^4^ The GRIPP checklist: strengthening the quality of patient and public involvement reporting in research	Provided a checklist for reporting studies on the impact of patient and public engagement in research
de Wit (2011)^27^ European League Against Rheumatism recommendations for the inclusion of patient representatives in scientific projects	Provided a list of eight recommendations for patient engagement in research projects
Ahmed (2010)^24^ Community engagement in research: frameworks for education and peer review	Provided two frameworks for community engagement in research
Hewlett (2006)^1^ Patients and professionals as research partners: challenges, practicalities and benefits	Provided the FIRST (facilitate, identify, respect, support, train) model to guide successful collaboration between patients and researchers

### 2.2 Data analysis

Data analysis was guided by Attride‐Stirling's six‐step thematic networks analytical technique to code and organize the data, and abstract, illustrate, describe and explore the emergent themes.^31^ This technique allowed us to focus on identifying concepts that form unique and interrelated themes of meaningful PEIR.^31^ Because the patient partners in the current project were APAB members, to protect the confidentiality of their APAB peers they did not review the full transcripts but were presented with critical excerpts from the interviews so they could question and confirm themes and contribute to the framework development. NVivo software (version 11, QSR International Pty Ltd, Burlington, MA) was used to manage the data.

**Figure 1:**
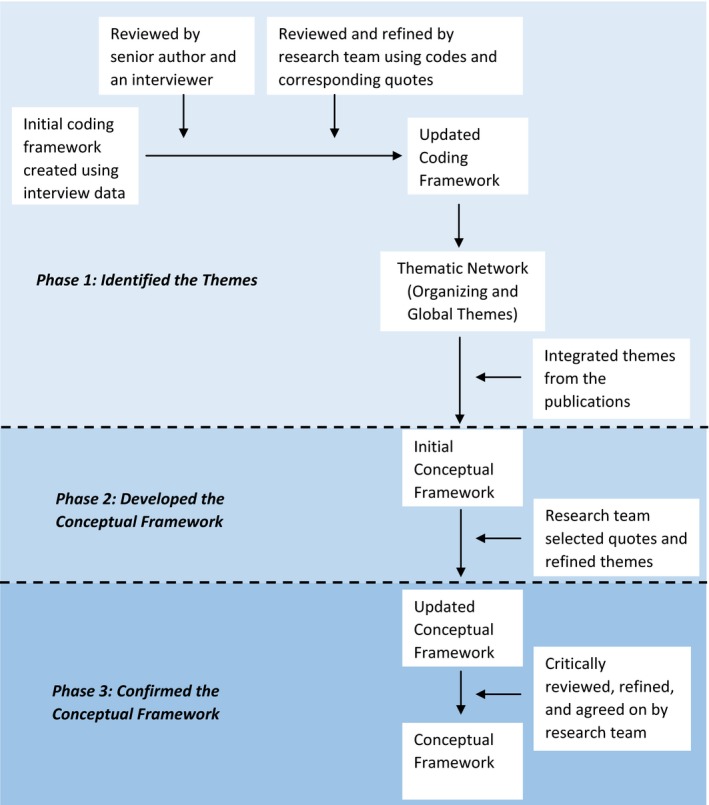
Summary of data analysis

The framework development involved three phases (Figure 1):

1. Identification of themes.
2. Development of the conceptual framework.
3. Confirmation of the conceptual framework.

#### 2.2.1 Phase 1: Identification of themes

This phase consisted of three steps: developing and applying a coding framework, abstracting the basic themes and abstracting the organizing and global themes. To develop the coding framework, eight transcripts were purposively selected based on participants’ APAB membership duration and total number of projects previously and currently engaged in. CBH and a research assistant read the full transcripts and conducted open coding. The resulting coding framework comprised 94 codes clustered into eight higher‐level codes, making the coding process more manageable.^31^, ^32^ Intercoder reliability for each of these eight codes was conducted by the two coders using the approach outlined by Campbell et al.^33^ A new transcript was then coded, and proportional agreement between the coders was calculated for each of the eight codes. Negotiated agreement was calculated when the 80% agreement threshold set for adequate intercoder reliability was not reached.^33^ These negotiations provided mutual understanding of each code by refining the code's definition and recoding disputed statements. This assessment was repeated with another new transcript to ensure acceptable levels of agreement. The reliable coding framework was then applied to all 18 transcripts by both coders, and any newly emergent codes were iteratively applied to all the transcripts.

After we incorporated feedback on our initial coding framework from LCL and one interviewer, other team members reviewed the refined coding framework via email. Discussions in a subsequent research team meeting led to further refinement of the codes and their definitions. The patient partners then reviewed the final coding framework to ensure it was comprehensive and reflected their experience as patient partners.^32^

Next, basic themes were created as statements by abstracting the central ideas in the portions of transcripts corresponding to lower‐level codes. The basic themes were then arranged into clusters of similar ideas and abstracted as organizing themes. The central idea across the organizing themes was abstracted into a global theme. The basic themes were the lowest order themes derived from the interview data, while organizing and global themes were successively higher‐order themes formed by clustering and abstracting the key concepts.^31^ The themes were then diagrammed as a thematic network, with the basic themes omitted to streamline the diagram.

Emergent themes were further validated via directed content analysis of the 18 key publications. Statements relevant to how to engage patients as research partners were extracted and coded by CBH. Subsequently, emergent themes from those publications were matched with organizing themes from the interview data. This helped to strengthen the descriptions of themes from the transcripts by affirming or refuting them and exploring interconnections between the experiences of our sample and those in published reports.

#### 2.2.2 Phase 2: Development of the conceptual framework

This phase involved two steps. CBH explored and interpreted the relationships among the organizing themes by comparing interview data and descriptions across the themes and assessing the relative importance, if any, of the themes.^32^ Our patient partners were then provided with several quotes from the transcripts and selected salient quotes corresponding to each organizing theme. CBH used these salient quotes to summarize the organizing themes and then compared the emergent conceptual framework with the existing guiding principles, frameworks, models and recommendations related to PEIR. This step allowed for broadening the interpretation of the conceptual framework beyond our interview data.^31^

#### 2.2.3 Phase 3: Confirmation of the conceptual framework

In this phase, all team members critically reviewed the framework. We refined the theme descriptions and documented our findings. Successive drafts of this paper were reviewed by all patient partners and researchers to ensure agreement on the presentation of the conceptual framework.

## 3 RESULTS

The 18 participants were aged between 26 and 68 (median 59) years, 17 were female, 14 had completed at least a college/university degree and most were either retired (*n*=7) or employed part‐time (*n*=4). They had belonged to APAB for between 1 month and 10 years, were involved in 0‐10 research projects (one was involved in zero) at Arthritis Research Canada and 12 had 1‐4 other chronic health conditions or diseases in addition to arthritis. The participants’ experiences of engaging in research alongside researchers were generally positive. The initial coding framework had adequate intercoder reliability (Supplementary 1). The phenomenon of meaningful PEIR from a patient perspective comprised of eight organizing themes, corresponding to the eight code clusters, which emerged from the interview data: *procedural requirements*,* convenience*,* contributions*,* support*,* team interaction*,* research environment*,* feel valued* and *benefits* (see Table 2 and Supplementary 2).

**Table 2:** Organizing themes of the PEIR Framework with examples of corresponding elements

Organizing themes	Example of elements
*Procedural requirements—*Procedural details involved in managing the inclusion of patient partners in a research project to ensure their experiences are rewarding and productive Overall, this theme highlights the importance of engaging early, sharing activities, having diverse patient partner representation, ensuring alignment between a research project and patient partners’ interests, having funding to support and compensate patient partners, being clear about the engagement process and having open communication	The research project has an appropriate number of patient partners Patient partners agree on the goals of the project Patient partners clearly understand their roles on the project
*Convenience*—Emphasizes the importance of choice and accessibility, including sufficient time to engage, and the flexibility to choose how and when to contribute	Patient partners have sufficient time to contribute Patient partners, preferences are considered when meetings are being planned
*Contributions*—Pertains to the roles of and tasks assumed by patients. Patient partners want to contribute their perspectives and experiences to research	Patient partners provide their perspectives The contributions are a good use of the patient partners’ time
*Team interaction*—Focuses on aspects of positive research team interaction that are important to patient partners, which involves communication style and rapport	There is mutual respect among team members Trust becomes established within the research team
*Research environment*—Emphasizes the importance of having a positive and an inclusive organizational/team culture that allows patients to feel comfortable and accepted as equal team members working together	Patient partners are treated as an equal partner There is a general openness to receiving the views of patient partners
*Support*—Pertains to the valuable resources, including financial and skills/instructional support offered to patient partners Highlights the importance of using financial and non‐financial resources to support and encourage patient partners’ contributions	Patient partners receive the training needed for their role Patient partners are offered sufficient reimbursement for out‐of‐pocket expenses
*Feel valued*—Focuses on ensuring that patients feel equally important on the research team by demonstrating appropriate recognition and respect	Patient partners contributions are acknowledged Patient partners are offered sufficient compensation for their contributions
*Benefits—*Highlights that it is important to patient partners that they derive benefits from their engagement	Patient partners see how their contributions can benefit other people Patient partners gain or improved their knowledge

### 3.1 Procedural requirements

“Procedural requirements” refers to the procedural details involved in managing the inclusion of patient partners in a research project to ensure their experiences are rewarding and productive. Participants wanted to contribute their experiences, knowledge and skills to projects that they found interesting, and wanted to be informed about the expectations associated with their roles as patient partners. One participant shared her thoughts on prerequisites for patient partners:

I think that critical thinking skills and like a desire to, an interest, and an ability to like explore… to think critically about things are really important. (Gemma)

Participants valued becoming gradually more engaged at their own pace:

I'm feeling a little more comfortable and so now it's just gradually I'm tackling a little bit more assignments and things. I'm starting to pick up more, more info and I'm taking a little more initiative to learn too and I've gotten more interested so that helps. (Laura)

Some specifically valued the use of lay language. For example, one participant stated:

Well, in the research I've been involved in, I've been very impressed with the effort to use layman terms instead of medical jargon or technical terms. The researchers bring it down to the patient level to explain it in words that we understand so I always found that really helpful. (Deka)

Furthermore, one participant spoke about the need for diversity among patient partners with regard to gender:

I think when there's the potential input into patient participation and research, if the organization is heavily female dominated, it's great for me but sort of the female issues on arthritis would be put forward.… It's one part and it's an important part but I think the perspective of males having arthritic conditions would also be important. (Susan)

Participants favoured making decisions as a team and using open communication that allowed knowledge exchange among team members. Receiving formal (ethics) approval as research team members enabled participants to undertake data collection and analysis and become more fully engaged in the research process. They wanted to be able to express their views without unnecessary restrictions. Some participants also spoke about wanting to be reimbursed and compensated (eg honoraria) for their contributions. Compensation would demonstrate that they are valued. Challenges relating to the scope of patient partners’ roles were also addressed. One participant highlighted differences in the roles of patient partners and researchers by noting the importance of researchers’ roles in maintaining the scientific quality of the research.

Analysis of the key publications corroborated these views. Specifically, publications including guiding principles on PEIR projects and systematic reviews indicated that projects should clearly outline roles, goals and expectations for team members at the outset.^6^, ^13^, ^15^, ^16^, ^24^, ^25^ Furthermore, the publications recommended that patient partners should engage continuously from a project's inception. This practice appeared to help patient partners become gradually more engaged and comfortable with the research process and environment.^30^ Two publications supported the use of plain language and minimization of undefined acronyms and technical terms.^24^, ^29^ Several publications also indicated that research projects should involve a diversity of patient perspectives, and that more than one patient partner should be invited to each project so that they could learn from and support each other.^13^, ^16^, ^23^, ^24^, ^25^, ^28^, ^29^ Projects should have a budget for engaging patient partners.^6^, ^30^ Two publications support distinct roles for researchers and patient partners, with researchers being responsible for maintaining scientific rigour.^11^, ^29^

### 3.2 Convenience

Participants valued convenience when engaging in research. This theme emphasizes the importance of choice and accessibility, including sufficient time to engage, and the flexibility to choose how and when to contribute, as exemplified in the following statements from two participants:

[P]eople who are involved have different medical conditions including arthritis… You know if they had a big exacerbation of their symptoms and need to pull back or pull out, that flexibility must be part of the [study] organization. (Susan)

[T]here had to be some flexibility of understanding that sometimes people can participate and sometimes they have to take breaks or, you know, there's different ability to participate. (Marie)

Participants valued the opportunity to access meetings remotely and were aware of the disadvantages of this approach:

The meetings that now can accommodate travelling and remote members, so that part's good and it gives us the opportunity to reach these other not so large metropolitan areas so I think we're just beginning to grasp that opportunity. In the future I would like to see that develop more but you've got to be careful because you have to have that meaningful patient engagement. (Julie)

The sentiments of participants concurred with the key publications, which support scheduling meetings at times convenient to patient partners and ensuring sufficient time for contributing, given their health and practical needs.^13^, ^23^, ^29^, ^30^ This might require research leaders to understand and take into account patients’ health‐related and practical restrictions to remove barriers and ease regulations to facilitate contributions from patient partners.^16^

### 3.3 Contributions

This theme pertains to the roles of and tasks assumed by patient partners. Participants expressed wanting to contribute their perspectives and experiences to research:

One way that I could help is to be involved in a research and impart my experiences and express my ideas and opinions to the researchers and hope that they listen and take into account what I went through as a legitimate contribution to their research. (Jan)

Participants’ contributions to research reflected the many dimensions of their lives and sometimes extended beyond sharing their health and health‐care experiences to include their professional and personal skills. For example, several participants spoke about assisting in the development of plain language summaries with researchers to disseminate research findings, which required critical thinking and communication skills. Some participants found constructive feedback important because sometimes they initially felt uncertain about their ability to contribute.

The concept of contributions was also addressed in the key publications. Specifically, one discussed the time required for patient partners to be involved in research activities and noted that there was no guarantee of a successful partnership or proper compensation.^26^ They corroborate the importance of giving patient partners constructive feedback on their contributions.^6^, ^10^, ^25^, ^30^

### 3.4 Support

Participants indicated that they received valuable financial and skills/instructional support. They valued financial support that covered their engagement‐related expenses, such as attending meetings. Skills/instructional support provided training to understand the language and processes of research. One participant shared her view on receiving training on data analysis:

[W]e may not do the statistics or that kind of thing but we may, it can help us to understand where the limitations of the study are and where, what is possible. (Marie)

The importance of financial support was corroborated by two of the 18 key publications.^6^, ^13^ Additionally, Cheung et al's^25^ recommendations for engaging patient partners on working groups highlighted the need for welfare support: “Counselors could be appointed to provide emotional support. Intimate and stable collaborations between a professional researcher and a [patient] research partner helped both to express their feelings and cope with tensions” (p. 193). Furthermore, de Wit et al^16^ noted that “According to the [patient] partners, not all [research] professionals provided the support required to sustain their motivation” (p. 497).

### 3.5 Team interaction

This theme highlighted the importance participants placed on positive research team interaction. Participants valued feeling comfortable and having enjoyable interactions when communicating with researchers:

All the researchers that I was involved with were amazing, amazing. They were very encouraging and very respectful. There's a fear when you start… However, the researchers and the research coordinators put you at ease immediately so you are able to relax and that enables you to speak and comment freely. (Lori)

Participants valued informal communication with other patient partners and connecting with the research team socially (eg research retreats or team lunches with researchers, research trainees and other patient partners):

[W]e have summer lunches and Christmas dinners, it's informal Christmas get together and just to talk about where're you going, what have you done, are you done with Christmas shopping things like what you would talk about with a close friend, kind of conversation. Yeah, lots of joking around, it's fun. (Jan)

Participants valued in‐person conversations with research team members, and researchers being accessible:

You just have to be comfortable relating with the researchers and because we have so many opportunities for face‐to‐face and I've been to his lab a couple of times and I chatted to him at different ARC [Arthritis Research Canada] events and so I'm comfortable to just zing it out there. (Julie)

The key publications corroborated that participants wanted a reciprocal relationship with other team members (researchers, research staff and patient partner)^16^ and valued positive interactions that establish mutual respect, trust and moral support.^9^, ^15^, ^26^ The importance of in‐person meetings was substantiated by the key publications.^23^, ^30^ For example, Forsythe et al^23^ wrote, “To establish strong working relationships, respondents [i.e., researchers who partner with patients and other stakeholders] recommended having multiple meetings and underscored the importance of face‐to‐face contact” (p. 19).

### 3.6 Research environment

This theme conveyed participants’ emphasis on the importance of having a positive and inclusive organizational/team culture that allows patient partners to feel comfortable and accepted as equal team members working together. Inclusivity is conveyed, for example, by providing equipment to enable patients to connect remotely and avoid commuting, and routinely inviting comments from all team members via phone or video, or in person. A positive and welcoming research environment fosters a feeling of connection with no emphasis on maintaining power or hierarchical differences among members:

He [the researcher] levelled the playing field so that we all felt very comfortable together and that was the biggest piece. If you don't feel comfortable then perhaps you won't raise your voice and ask questions or comment because you might not feel that what you have to say is worthy but [researcher], nope, brought everyone together. He was the backbone of the group. He made sure that we were included in everything. (Phoebe‐Lewis)

Consistent with this theme, one publication suggests that a safe environment may help to promote contributions by patient partners.^10^

### 3.7 Feel valued

Participants expressed wanting to feel equally important on the research team, with appropriate recognition and respect demonstrated through, for example, compensation for their contributions. One participant explained:

It's not that I want to personally make money doing this… It's respect, yeah, because if they're not paying for your opinion, it doesn't mean much to them. It's like disposable. (Hannah)

Furthermore, they wanted their views to be recognized and used:

I would have to say respect for the patient's viewpoint. That was most important because if there wasn't any respect for that viewpoint, there'd be no point in us being there. So don't ask us to the table if you are going to dismiss what we have to say. (Phoebe‐Lewis)

This theme was a common concept in the key publications.^9^, ^16^, ^25^, ^30^ For example, one guideline recommended, “Do something with the input given by the partners. Listening only is not enough.”^30^ Three of the key publications also recommended compensating patient partners as a means of acknowledging them.^6^, ^25^, ^30^

### 3.8 Benefits

It was important to participants that they benefitted from their engagement. They spoke about personal benefits such as feeling empowered through gaining confidence, knowledge and skills to communicate their perspective in a research team. In addition, most participants expressed that learning about their diseases and treatments was important, as well as learning in general through their work with researchers:

Over the last five or six years I've been involved in research for arthritis. I found it very informative. When I was first diagnosed, I found actually it was very helpful for me to learn about my disease, and the impact of patients expressing their views and talking to researchers and health care providers and figuring out what worked for them. So, I've always found when I've been involved in the research I've learned something to help me. (Deka)

For some participants, engaging in research provided renewed purpose and led to a positive change in their lives:

An arthritis diagnosis can make you feel powerless but collaborating with researchers that listen to and appreciate your feedback gives you some of that power back; personally, it made me feel as though I was contributing to finding answers for people with this disease. (Lori)

Furthermore, participants valued helping others, influencing decisions and contributing to evidence‐based solutions:

I'm happy to contribute and be part of the solution that could help others who are suffering with the disease. It's [a] very fulfilling feeling. (Jan)

The key publications highlighted that patient partners engaged when benefits outweighed the risks and that patient partners should learn how to engage in research.^6^, ^16^, ^19^, ^24^, ^29^, ^30^ Also, Ahmed et al^24^ highlighted the role of learning in building capacity among researcher or patient communities: “Investigators and the community partner to learn from each other and share expertise and knowledge” (p. 1382).

### 3.9 Patient engagement in research: A conceptual framework

These eight organizing themes formed the global theme *Meaningful PEIR*. Meaningful PEIR is the planned, supported and valued involvement of patients in the research process within an interactive team and positive research environment that facilitates effective contributions by patients or their surrogates to help to produce important outcomes while benefitting the patients or their surrogates. The data suggest interconnections among the eight organizing themes over the course of engagement, but our analysis did not conclusively map these interconnections and the cause and effect relationships. For example, patient partners’ contributions could be facilitated when researchers maintain certain procedural requirements, offer opportunities to engage in ways that patient partners find convenient and provide adequate support, such that patient partners feel valued and identify benefits from engaging with a research team. Offering compensation to patient partners was a Procedural Requirement and made patient partners Feel Valued. Feeling valued seems related to having an inclusive research environment, positive team interactions and support. Some Benefits to patient partners overlapped with aspects of Support, such as accessing scientific articles, and with aspects of Team Interaction, such as interacting with peers. As one example of interconnections across the concepts, respect appeared to underlie Support, Feel Valued and Team Interaction. However, further research is necessary to confirm the PEIR Framework concepts and map how they relate to one another.

## 4 DISCUSSION

This study revealed eight themes that underscore what patient partners valued as important to PEIR. This study is among the first to use empirical data from a sample of patient partners to provide a conceptualization of meaningful PEIR. The results expand on current understanding in the literature by providing a conceptual framework based on a patient perspective.

The PEIR Framework extends beyond the interviews by incorporating, streamlining and orienting published attributes of PEIR towards a patient perspective.^1^, ^4^, ^6^, ^9^, ^10^, ^11^, ^13^, ^15^, ^16^, ^19^, ^23^, ^24^, ^25^, ^26^, ^27^, ^28^, ^29^, ^30^ The Canadian Institutes of Health Research^10^ and Patient‐Centered Outcomes Research Institute^9^ provided guiding principles that are beliefs about and core values of engaging patients in research. However, the PEIR Framework extends beyond this by offering some practical details (Table 2). Compared to the PEIR Framework, one important concept not emphasized in the facilitate, identify, respect, support, trust (FIRST) model is the personal benefits to patient partners, such as learning about their disease.^16^ The FIRST model instead emphasized identifying projects that would benefit from patient partners’ contributions and indicated that when patient partners learn, the project ultimately benefits.^16^ The summary of existing guiding principles of patient engagement by Kirwan et al^13^ also does not highlight benefits to patients beyond co‐learning. Soever et al^19^ reported that learning from research team members was a reason for their participants’ engagement in research, but did not include it in their “conceptual framework for interrelational health‐care research.”

To our knowledge, the PEIR Framework is the first framework for PEIR to use empirical data derived exclusively from a patient perspective. Soever et al developed their conceptual framework from the combined perspectives of professional researchers/clinicians, stakeholder group personnel and two patients. Conversely, we privileged a patient perspective because our primary data source is patients, supplemented by trends in the literature. Soever et al's framework has two broader themes divided into nine more nuanced themes: structural prerequisites (team leadership, expertise in the topic area, organized/coordinate management, funding, time/associated workload and forum for multimodal communication) and values (trust, common interests and respect for each other). The PEIR Framework covers those themes, but does not directly align with their scope and descriptions. For example, common interest(s) in Soever et al's framework denote positively impacting the outcome of the research.^19^ The idea of “common interest(s)” is reflected in our procedural requirements for having the research topic and patients’ interests aligned, and patients having the capacity to contribute. Both frameworks present comparable ideas. However, a key difference is that the patient perspective is a defining feature of the PEIR Framework. The PEIR Framework therefore orientates the reader to what is important to patient partners for meaningful PEIR to be achieved.

### 4.1 Practical value

The PEIR Framework (Table 2) could be applied by using its eight themes to guide the development of an engagement plan. The specific elements of the themes can be co‐constructed and agreed on by the research team members. These elements would then guide PEIR and be updated when necessary. The elements could form items to evaluate the extent to which meaningful PEIR was accomplished.

### 4.2 Limitations

Participants from the interviews lacked diversity in gender, education and diagnosis (all had arthritis as a primary diagnosis), and did not include informal caregivers (eg family members and friends) of patients. Our participants’ views primarily reflected the context of engagement of patients through an APAB and research project teams led or co‐led by researchers at a single research centre for arthritis research. This framework therefore might not reflect other contexts of PEIR, such as patient‐directed research. The PEIR Framework does, however, reflect the rich diversity of experiences and views of our participants who had experiences with varying degrees of PEIR, and so could be relevant to other patient communities as many of the concepts were also reported in the key publications.

### 4.3 Next steps

We speculate that there are both common and unique elements of meaningful PEIR across demographic characteristics of patient partners. The PEIR Framework could be studied with a demographically diverse sample, to further elucidate its general structure and nuances across patient communities, and to determine elements of each theme that are applicable for planning, implementing and evaluating PEIR in various contexts. For example, the level and type of support needed might vary by research experience or education level of patient partners. Further, the PEIR Framework is currently being used to develop an outcome measure for this phenomenon.

## 5 CONCLUSIONS

This novel conceptual framework can be used to guide teams in developing strategies for engaging patients in research activities. Our findings specifically offer an orientation on what is important to patient partners, including deriving benefits and feeling valued. The PEIR Framework could be particularly useful when patient‐researcher partnerships are led by researchers with little experience of engaging patients in research, but it needs further validation.

## CONFLICT OF INTEREST

The authors have no conflict of interest to declare.
