# Supplementary material for: An empirically based conceptual framework for fostering meaningful patient engagement in research
Source: Health Expect. 2017 Oct 6;21(1):396–406. doi: 10.1111/hex.12635 (PMC5750689; doi:10.1111/hex.12635)
Supplement: Supplementary file 1 [file HEX-21-396-s001.docx]

| **Supplementary File 1**  **Result of Intercoder Reliability Assessment** | | | | | |
| --- | --- | --- | --- | --- | --- |
| **Code** | **Description** | **Round 1*** | | **Round 2*** | |
|  |  | Proportional Agreement, % | Negotiated Agreement, % | Proportional Agreement, % | Negotiated Agreement, % |
| Adapt to PRP | The opportunities for PRPs to engage are unrestricted and fit their preferences. | 80 | 100 | 87 | ND |
| Benefits to PRP | The tangible benefits obtained by PRPs because of their engagement in the research process. | 52 | 100 | 86 | ND |
| Tasks and Roles | Tasks and roles PRPs performed or expected to perform when engaging in research, including the use of their time. | 93 | 93 | 83 | ND |
| Patients Feel Valued | Feel that their contributions have been recognized and used, and that they are respected. | 79 | 100 | 100 | ND |
| Procedural considerations | The procedural details involved in managing the inclusion of PRPs in a research project. | 40 | 82 | 81 | ND |
| Research Environment | This pertains to the nature of the research setting, such as its culture. | 55 | 100 | 69 | 100 |
| Research Team Interaction | Captures the patient’s views and experiences regarding interacting with research team members. | 49 | 100 | 100 | ND |
| Support | Financial and non-financial resources provided to patient to compensate or encourage his/her participation. | 69 | 100 | 65 | 100 |
| PRP = Patient Research Partner; ND = Not done because it obtained the 80% threshold for proportional agreement  *Values of > 80% were interpreted as adequate intercoder reliability. | | | | | |
