# Supplementary material for: An empirically based conceptual framework for fostering meaningful patient engagement in research
Source: Health Expect. 2017 Oct 6;21(1):396–406. doi: 10.1111/hex.12635 (PMC5750689; doi:10.1111/hex.12635)
Supplement: Supplementary file 2 [file HEX-21-396-s002.docx]

| **Supplementary File 2**  **Themes demonstrating the PRPs experiences and views of about engaging in research** | |
| --- | --- |
|  | **Quotations from participants** |
| Procedural Requirements | I don’t attend the meetings anymore but when I did attend meetings, the most important part for me was to have the agenda beforehand. (Lori)  The first meeting was actually an introductory meeting so that it was like an interview if you like where I sat through a portion of their meeting to get a, an idea of what goes on at those meetings…. And so that I would get a sense of the responsibilities and you know the expectations and all of that, but also if our personalities meshed and if I had skills that were needed to kind of complement the team that they’d already put together. (Madeline)  I think that we want to represent the patient voice and patients come from every socioeconomic bracket and I think it’s important that we’re able to provide opportunities for learning or engagement with patients from every different socioeconomic bracket and if we talk in a language that is unattainable, we will never reach that goal. (Julie) |
| Convenience | Because I travel so much, I would join a meeting by Skype or we had one member, APAB MEMBER 8, LOCATION 2, she would join by Skype but the quality was horrible. It was hard to hear, fuzzy, connections were lost, things like that and then over time, it was suggested that we look at better video conferencing systems. (Julie)  And I have done, in my work I’ve, in the past, I’ve done a lot of teleconference and video conferencing so I’m actually comfortable with doing that. But it is, it’s hard because you aren’t necessarily, because you’re not there sometimes you, you don’t become part of a conversation, it’s more difficult to get into the conversation. It requires the people who are leading the meeting, chairing the meeting to actually make a point of making sure that you’re part of it. (Marie)  Everyone here really understands that these people live with certain restrictions on how much they can take on and for me I don’t even know if that’s illness related or if it’s just a human can only do so much. (Olivia)  Sitting that long, my hip starts to bother me. Actually in our last meeting a couple of weeks ago, I had to stand after one and a half hour. The good thing is it’s flexible and I’m not the only [one] standing. At least half of us are having our stretches in between. (Jan) |
| Contributions | Everyone contributes in different ways and a lot of that is like what kind of ties that altogether is like the kind of respect for the diversity that’s there and the diversity of skill sets and letting people do kind of what they, what they want to and feel comfortable with, kind of within the general framework of things that get done. (Gemma)  I just got involved in some of the plain language summaries this week, reviewing them and that was really interesting as well because I must be able to look at a lot of different research that’s happened and see how easy it is to break down all of that complex information into 200 to 400 words. (Chloe)  I like getting feedback after the fact to let me know how the study proceeded or what was the outcome. (Julie)  Ya, you know, the thing that really amazed me and impressed me about APAB when I first started up was wow these people all have arthritis to varying degrees, they all have, you know, various impairments and they are doing so much, they’re accomplishing so much on this board. I was so utterly impressed. I’ve never seen such a like mobilized group of volunteers achieve so much,… (Norma) |
| Support | I really couldn’t commit the time to do the work that I needed to and so the group was very good about allowing me to take a bit of time off. I had other people to cover for some things that were important and then I was able to come back and [resume] those duties. So we take care to help each other out and they encouraged me to come back. It wasn’t something where oh you can’t do this so maybe you should quit. It was, you know, we want to welcome you back when you’re ready. (Marie)  And others that have just come up and just been very friendly and um wanting to make sure that I wasn’t too overwhelmed and letting me know that you know it will get a lot easier and the info will be a lot easier to understand and the different activities will become clearer in the future and that if I am unclear about anything that I can definitely ask anyone. (Chloe)  I mean we don’t have to eat dinner at 4 o’clock or go to a meeting at 7. Instead at 5 o’clock that’s one of the big things is being able to have a meal there [at APAB monthly meeting], so that you aren’t really late. (Victoria) |
| Team Interactions | Yeah, so yeah, we don’t always make the mark, sometimes we get off track a bit but from my perspective…at the top of the [agenda] page it should always be, “It’s about the research” and it’s about patients in research, patients’ interaction with the researchers, so the engagement is more than engagement, it’s interaction I think. (Julie)  For example, I remember e-mailing back and forth with him as early as 5 o’clock in the morning and as late as midnight; he was so very accessible. (Lori)  I do communicate with two or three [APAB] members via email more closely than I do with other people. Um, I have things in common with, with some members and we talk about the things that we have in common, the things that we enjoy. (David) |
| Research Environment | One of the things that my initial observation is that people are really friendly, very committed and interested, and certainly inclusive towards me being a newcomer. (Susan)  At ARC [Arthritis Research Canada] APAB is embedded within ARC which means that there’s an ongoing relationship through many events whether they be social events or whether they be fundraising events or whether they be research events. There’s an ongoing relationship that enables more in-depth partnership that isn’t available, to my knowledge, in other academic research pursuits that involve patients. (Jessica)  I don’t see a single person in ARC that has an, “I am God. I am the most important. I am the director. I am the head of this group and everybody on this group is going to say what I want them to say.” (Victoria) |
| Feel Valued | And I think that that’s really important because patients are going to be reticent to contribute if they feel it’s tokenism. If they feel it’s really valued then that’s going to encourage them to speak and contribute more freely without fear that they should only say something that they think, you know, that they wouldn’t remain silent if there was an opportunity to present an idea even if it was really far out. (Jessica)  My expectation is that my input would be seen as important and valuable. I think just having patients involved in research means that the researchers are amenable to that, you know that because they would agree to have patients involved, obviously they think that that’s important. (Susan)  I don’t need any accolades or anything like that, that’s not why I’m doing what I’m doing, you know, but I find that, I think when people feel appreciated they’re willing to even do more and so I think that’s with anything that you do. If there’s an appreciation, you know, that your voice matters, that your input matters and yes we hear you, I think that’s really important. (Laura)  So there have been some research projects where our feedback has made a difference in how the research was designed just from some of the questions and comments that we had the researchers took that and actually said oh, ya, let’s, we can look at this, we can make some changes here. So to me that’s showing respect to the patient perspective. (Marie) |
| Benefits | I got brought into APAB and I really liked the people and I liked that it was connected to a research organization so I just stayed and more opportunities came. (Olivia)  But in opening doors for us so that we could have the opportunity to submit an abstract, have it reviewed and considered for conferences. We attended many [conferences]. RESEARCHER 7 opened a door for APAB MEMBER 1 to become a member of the OMERACT group by pointing her in the right direction and to the right person. (Phoebe-Lewis)  I could quote what my mum said, “When you joined that group, all of a sudden you had a purpose again”, not just my hobbies and my things that I do but I all of a sudden had a purpose but also I have a whole group of friends here. (Victoria)  Over the last 10 years, the researchers at ARC have benefitted from the input that clients have given them and now it’s a symbiotic kind of relationship where we also see, us consumers, the benefit of spending time to give our thoughts to the researchers because it will help them be interested in areas that we want them to be interested in. (Madeline) |
